# Sexual dimorphism of microglia and synapses during mouse postnatal development

**DOI:** 10.1002/dneu.22568

**Published:** 2018-01-04

**Authors:** Laetitia Weinhard, Urte Neniskyte, Auguste Vadisiute, Giulia di Bartolomei, Nil Aygün, Laurie Riviere, Francesca Zonfrillo, Susan Dymecki, Cornelius Gross

**Affiliations:** ^1^ Epigenetics and Neurobiology Unit, European Molecular Biology Laboratory (EMBL) Monterotondo Italy; ^2^ Department of Genetics Harvard Medical School Boston Massachusetts; ^3^ Present address: Life Science Center, Vilnius University Vilnius Lithuania

**Keywords:** sex differences, microglia, hippocampus, synapse formation, postnatal development

## Abstract

Microglia participate in synapse remodeling in the cortex and hippocampus during mouse postnatal development. Although sex differences in microglia activity during embryonic development have been reported in these regions, it remains unexplored whether microglia show sexually dimorphic features during the early postnatal period, a critical window for synapse formation and maturation. Here, we investigated morphological and functional features of microglia across early postnatal development as well as morphological features of both pre‐ and postsynaptic neuronal compartments in the mouse hippocampus. We found a sex‐dependent shift in microglia volume and phagocytic capacity across the first four postnatal weeks. Measurements of synaptic features revealed sex differences in the density of synaptic spines and boutons during the second postnatal week. These data are consistent with a precocious development of both microglia and synapses in the female brain. We further hypothesize that this bias may contribute to sex‐specific brain wiring. © 2017 The Authors. Developmental Neurobiology Published by Wiley Periodicals, Inc. Develop Neurobiol 78: 618–626, 2018

## INTRODUCTION

Microglia are the resident immune cells of the brain. They are produced in the yolk sac during the earliest phase of hematopoiesis (Ginhoux et al., 2010) and colonize the developing neuroectoderm before the blood‐brain barrier forms. They subsequently proliferate and mature from an amoeboid, phagocytic type to a ramified, surveilling state (Dalmau et al., 1998, Wake et al., 2009). As a result of the plethora of receptors they express to sense their environment, microglia are highly sensitive to any disturbance of brain homeostasis (Kettenmann et al., 2011). Microglia have been recently demonstrated to play an important role in synaptic circuit remodeling through the formation (Parkhurst et al., 2013; Miyamoto et al., 2016), elimination (Tremblay et al. 2010; Paolicelli et al., 2011; Schafer et al., 2012), and maturation of synapses (Hoshiko et al., 2012; Zhan et al., 2014; Sipe et al., 2016). The importance of microglia in brain development was further demonstrated in several studies that associated aberrant microglia function with neurodevelopmental diseases such as autism and schizophrenia (Tetreault et al., 2012; Zhan et al., 2014; Sekar et al., 2016; Werling et al., 2016). Both of these neuropsychiatric conditions show a strong sex bias, with higher prevalence in males (McGrath et al., 2004; Werling and Geschwind, 2013). Intriguingly, microglia were shown to be involved in sexual differentiation of brain regions that control sexually dimorphic behaviors (Lenz et al., 2013), while sex differences in microglia morphology and function were recently reported in the mouse brain at perinatal (Schwarz et al., 2012; Nelson et al., 2017) and late postnatal stages (Schwarz et al., 2012; Hanamsagar et al., 2017). However, no sex differences in microglia have been reported in the first postnatal weeks, a critical time‐window for synaptic circuit formation and maturation, and it remains unknown if any such sex differences could influence hippocampal circuit development. We therefore investigated microglia morphology across postnatal stages together with the morphology and density of pre‐ and postsynaptic structures to identify sexual dimorphism in microglia and synapse development in the postnatal hippocampus. We observed a transient peak in microglia volume and CD68 immunocolocalization, a measure of phagocytic capacity, during the second postnatal week in both sexes. However, microglia volume and phagocytic capacity increased and decreased earlier in females than males. This shift was paralleled by a difference in the density of neuronal spines and boutons. The pattern of differences in microglia and synaptic features is consistent with a precocious development of microglia and synapses in the female brain.

## MATERIALS AND METHODS

### Animals

**Figure 1:**
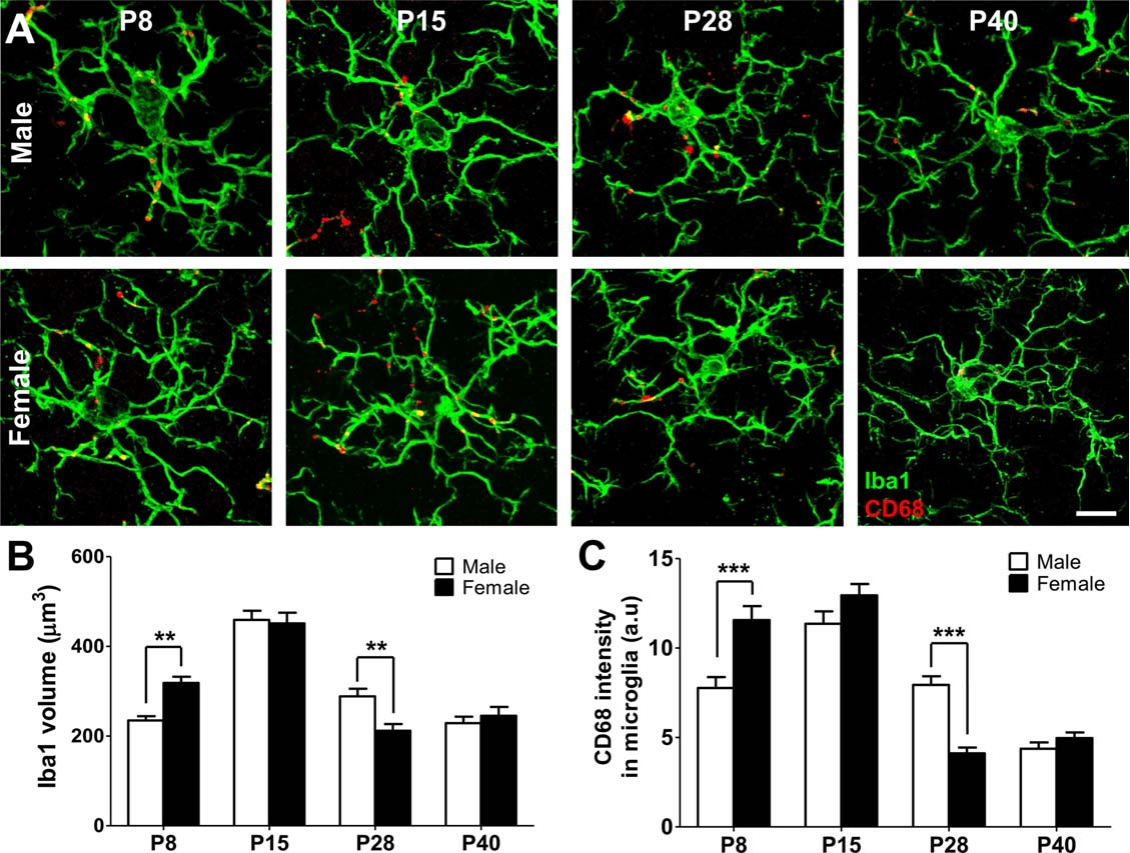
Sex differences in microglia volume and phagocytic capacity across postnatal development. (**A**) Representative images of Iba1 immunolabelled microglia (green) containing phagocytic compartments immunostained for CD68 (red) at P8, P15, P28, and P40 in the male and female CA1 region of the hippocampus. (**B**) Microglia size was assessed as Iba1+ volume (*n* = 60 cells from three animals for both sexes at each timepoint). (**C**) Microglia phagocytic activity was estimated using colocalization of Iba1/CD68 immunofluorescence (*n* = 60 cells from three animals for both sexes at each timepoint). Data are presented as mean ± SEM, ***p < 0.001, two‐way ANOVA. Scale bar = 10 µm. [Color figure can be viewed at http://wileyonlinelibrary.com]

**Figure 2:**
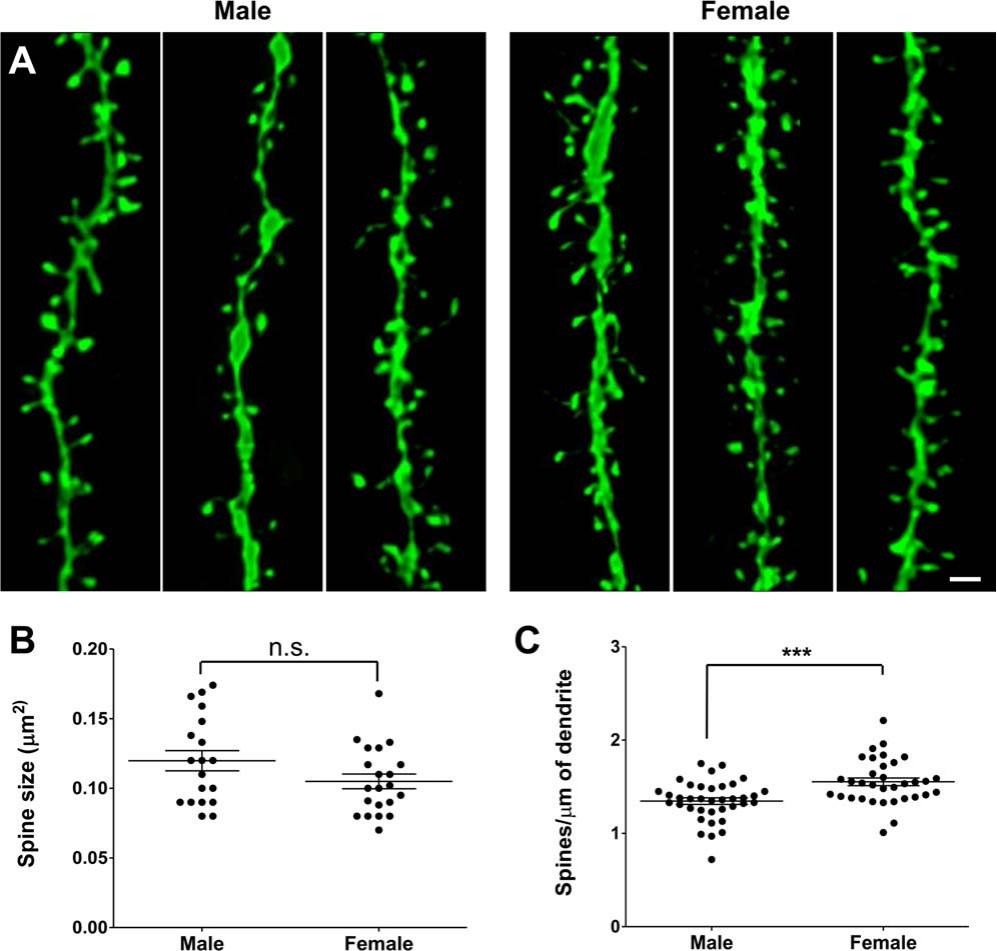
Increased synaptic spine density in females at P15. (**A**) Representative images of GFP+ secondary dendrites in the CA1 region of the hippocampus in *Thy1*::GFP males and females at P15. (**B**) Spine size measurement revealed no difference between males and females (*n* = 19 and 21 neurons, respectively, from four animals for both sexes). (**C**) Spine quantification revealed a higher density in females compared to males (*n* = 34 and 37 neurons, respectively, from four animals for both sexes). The data are presented as mean ± SEM, *** *p* < 0.001, *t*‐test. Scale bar = 1 µm. [Color figure can be viewed at http://wileyonlinelibrary.com]

**Figure 3:**
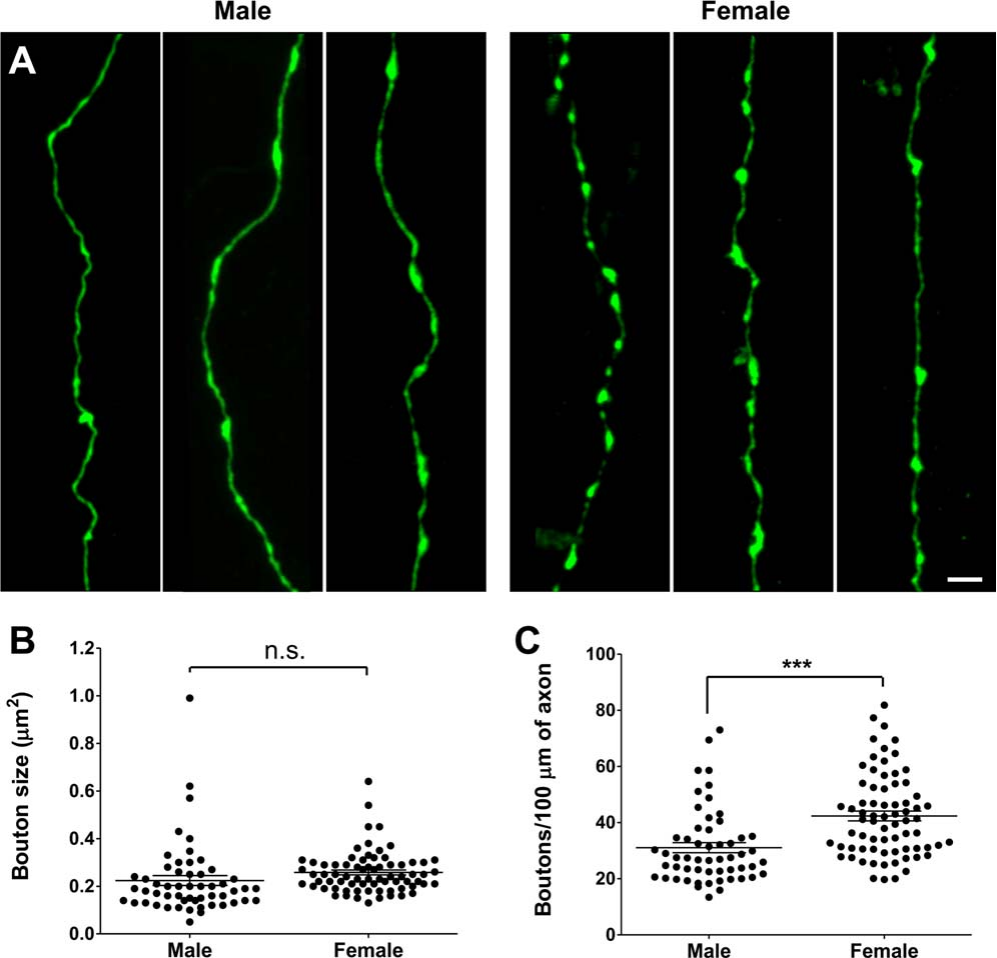
Increased synaptic bouton density in females at P15. (**A**) Representative images of GFP+ CA3‐CA1 axons in *Thy1*::GFP males and females at P15. (**B**) Synaptic bouton size measurement revealed no difference between males and females (*n* = 55 and 72 neurons from four and five animals, respectively). (**C**) Synaptic bouton quantification revealed a higher density in females compared to males (*n* = 72 and 55 neurons from five and four animals, respectively). The data are presented as mean ± SEM, *** *p* < 0.001, *t*‐test. Scale bar = 2 µm. [Color figure can be viewed at http://wileyonlinelibrary.com]

All mice were maintained on a C57BL/6J congenic background. Mice were bred, genotyped and tested at EMBL following protocols approved by the EMBL Ethics Committee and the Italian Ministry of Health. Homozygous *Thy1*::EGFP‐M (Feng et al. 2000, Jackson Laboratory stock #007788) were used for dendritic spine and axonal bouton analysis. Heterozygous *Emx1*::Cre; *RC*::PSiT littermates were used for synapse density measurements. The *RC*::PSiT allele was obtained by crossing the dual Cre/Flp‐dependent *Rosa26*‐*CAG*::FSF‐LSL‐SynaptophysinGFP‐ires‐tdTomato‐WPRE allele (Niederkofler et al., 2016) with a Flp deleter allele (Dymecki, 1996) to permanently remove the FRT‐flanked STOP cassette. Crossing of these Cre‐dependent Synaptophysin‐GFP reporter mice to the *Emx1*::Cre driver line (Iwasato et al., 2004, with FRT‐flanked neo cassette removed) resulted in systematic labeling of cortical excitatory neuron boutons. To visualize synaptophysin‐GFP in hippocampal excitatory pyramidal neurons homozygous *RC*::PSiT males were bred to heterozygous *Emx1*::Cre females. In order to minimize the number of animals used, some data in Figures 1, 2, 3 comes from mice that were not littermates. Subsequent analysis of that part of the data strictly deriving from littermates confirmed all phenotypic differences reported (data not shown). In addition, we ruled out a possible confound due to differences in estrus status across female mice by demonstrating that the between‐animal variability in data from females was not significantly different from that of littermate males (data not shown).

### Microglial Morphology and Immunocolocalization Analysis

Wild‐type mice were anesthetized intraperitoneally with 2.5% Avertin (Sigma‐Aldrich, St Louis) and perfused transcardially with 4% paraformaldehyde (PFA) at P8, P15, P28, and P40. Brains were removed and post‐fixed in 4% PFA overnight at 4°C. Coronal 50 µm sections were cut on a vibratom (Leica Microsystems, Wetzlar, Germany) and blocked in 20% normal goat serum and 0.4% Triton X‐100 in PBS for 2 h at room temperature. CD68 and Iba1 were immunodetected by overnight incubation at 4°C with primary antibodies (rat anti‐CD68 1:500, AbD Serotec; rabbit anti‐Iba1 1:200, Wako) followed by secondary antibodies (goat anti‐rat A546 and goat anti‐rabbit A647, 1:400, Life Technologies) incubation in PBS with 0.3% Triton‐X100 and 5% goat serum for 2 h at room temperature. The *stratum radiatum* of the hippocampal CA1 region was then imaged on a SP5 resonant scanner confocal microscope (TCS Leica Microsystems, Mannheim, Germany) with a 63x/1.4 oil immersion objective at 48 nm lateral pixel size with an axial step of 130 nm. Microglia Iba1 volume and CD68 signal intensity were measured with Imaris software (Bitplane, Zurich, Switzerland) in individual cells after 3D reconstruction using local contrast.

### Synaptic Spine Size and Density Quantification

Brain sections from P15 and P40 *Thy1*::EGFP animals were obtained as previously described. Bright GFP+ dendrites were imaged in the *stratum radiatum* of the hippocampal CA1 region with a SP5 resonant scanner confocal microscope with a 63×/1.4 oil immersion objective at 40 nm lateral pixel size with an axial step of 130 nm. Images were deconvolved using Huygens software (40 iterations, 0.1 of quality change, theoretical point spread function) and sharpened using Image J software (NIH). For semi‐automatic quantification of spines and measurement of their head size, dendrites were axially projected as maximum intensity on Image J so only lateral spines were analyzed. Signal intensity was measured in the dendritic shaft and normalized across all datasets. Noise signal was measured outside the dendritic shaft and removed by adjusting the minimum to the value measured + 40%. Automatic thresholding was subsequently applied using the Huang algorithm, upon exclusion of shaft and neck signal. Spine number and size were subsequently analyzed using particle measurement, after sphericity (>0.3/1) and size (>0.005 μm^2^) thresholding to avoid false positives. For each neuron, a total dendrite length of 100–150 μm from three different secondary dendritic branches was analyzed.

### Synaptic Bouton Size and Density Quantification

Brain sections from P15 and P40 *Thy1*::EGFP animals were obtained as previously described. Bright GFP+ axons were imaged in the *stratum radiatum* of the hippocampal CA1 region with a SP5 resonant scanner confocal microscope with a 63x/1.4 oil immersion objective at 60 nm lateral pixel size with an axial step of 300 nm. For semi‐automatic quantification of boutons and the measurement of their size, GFP+ axons were axially projected as maximum intensity on Image J. Signal intensity was measured in the axonal shaft and normalized across all datasets. Boutons were discriminated from the axonal shaft by adjusting the minimum to the signal measured in the shaft +40% (Becker et al. 2008). Bouton number and size were subsequently analyzed using particle measurement, after sphericity (>0.3/1) and size (>0.01 μm^2^) thresholding to avoid false positives. For each neuron, a total axonal length of 100–300 μm was analyzed.

## Synapse Density Quantification

Brain sections from P15 *Emx1*::Cre;*RC*::PSiT littermates were obtained as previously described. The *stratum radiatum* of the hippocampal CA1 region was imaged on a SP5 resonant scanner confocal microscope with a 63x/1.4 oil immersion objective at 35 nm lateral pixel size with an axial step of 130 nm. For each hippocampal slice, volumes of 70 × 35 × 2 μm containing no nucleus or vessel were selected for signal intensity measurement and synapse density analysis using Imaris.

## RESULTS

### Sex Differences in Microglia Morphology and Phagocytic Capacity

In order to investigate sexual dimorphism in microglia we measured microglia volume and phagocytic activity from early to late postnatal stages in the hippocampus of male and female mice. We performed volume measurements of Iba1‐immunolabelled microglia, and immunocolocalization analysis of microglial cytoplasm with the phagosomal marker CD68 in fixed hippocampus sections at postnatal day 8, 15, 28, and 40 (P8, P15, P28, P40; Fig. 1). Our analysis revealed that microglia volume and CD68 colocalization peaked at P15 and declined at P28 and P40. At P8 females showed significantly increased microglia volume (309 ± 13 μm^3^ vs. 236 ± 9 μm^3^, ***p < 0.001, two‐way ANOVA, *n* = 60 cells from three animals per sex group) and significantly increased CD68 colocalization (11.0 ± 0.7 a.u. vs. 8.0 ± 0.6 a.u., ***p < 0.001, two‐way ANOVA, *n* = 60 cells from three animals per sex group) when compared to males. While at P15 no significant differences were detected, P28 females showed significantly decreased microglia volume (293 ± 16 μm3 vs. 216 ± 14 μm3, ***p < 0.001, two‐way ANOVA, *n* = 60 cells from three animals per sex group) and CD68 colocalization (7.9 ± 0.4 a.u. vs. 4.3 ± 0.3 a.u., ***p < 0.001, two‐way ANOVA, *n* = 60 cells from three animals per sex group) when compared to males. Altogether, our data revealed a sex‐dependent shift in the developmental trajectory of microglia morphology and phagocytic capacity.

### Sex Differences in Synapse Density during Early Postnatal Development

**Figure 4:**
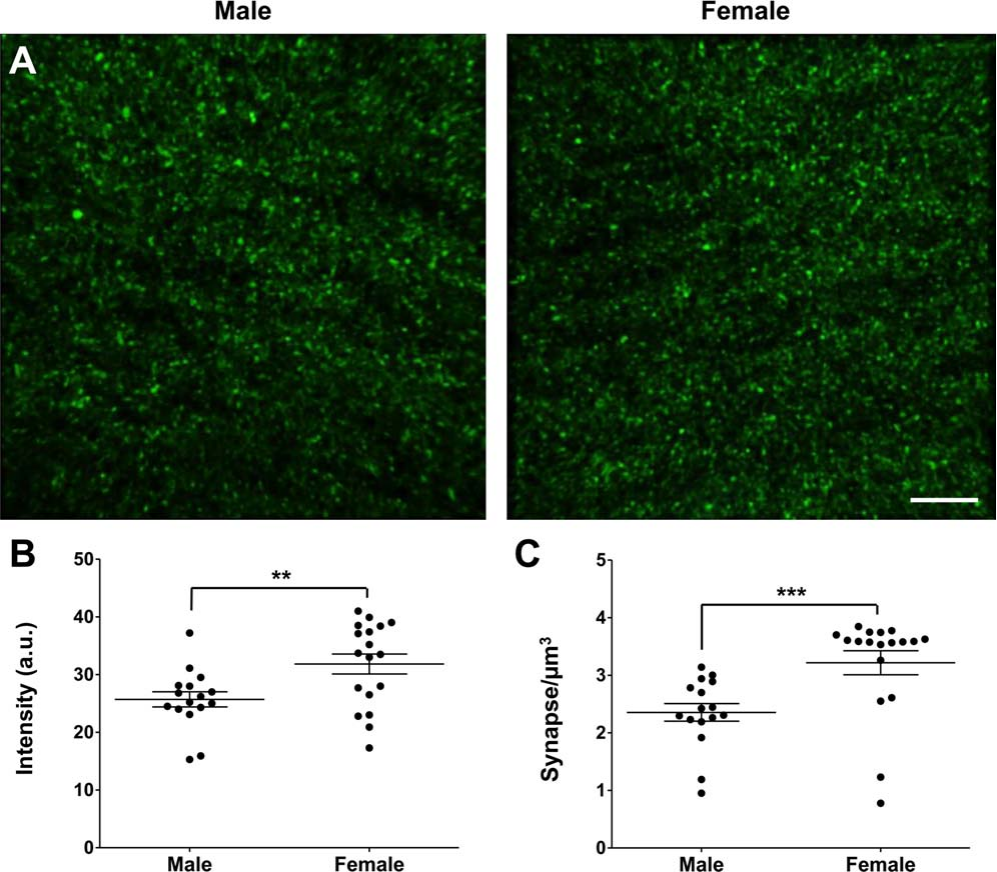
Increased functional synapse density in females at P15. (**A**) Representative images of the CA1 region of *Emx1*::Cre; *RC*::PSiT male and female at P15. (**B, C**) Intensity (**B**) and number (**C**) of synaptophysin‐GFP puncta revealed a higher density of synaptophysin‐containing synapses in females compared to males (*n* = 18 and 16 hippocampus analyzed, respectively, from four animals for both sexes). The data are presented as mean ± SEM, **p < 0.01, ***p < 0.001, *t*‐test. Scale bar = 5 µm. [Color figure can be viewed at http://wileyonlinelibrary.com]

Microglia have been shown to engulf synaptic material, and mutations that disrupt neuron‐microglia signaling are associated with decreased pruning of synapses during development (Paolicelli et al., 2011; Schafer et al., 2012). On the other hand, microglia have been shown to induce the formation of neuronal filopodia and new functional synapses in the cortex (Parkhurst et al., 2013; Miyamoto et al., 2016), suggesting that microglia may be involved in both synapse removal and formation during circuit assembly and homeostasis. To explore whether sex differences in microglia might be accompanied by differences in synapses, we used the *Thy1*::GFP reporter to perform a morphological analysis of both pre‐ and postsynaptic structures at P15, when microglia volume and phagocytic capacity was found to be maximal. We observed no difference in spine size between males and females (0.12 ± 0.007 vs. 0.11 ± 0.005 µm^2^, respectively, *p* = 0.1, *t*‐test; [Fig. 2(A,B)]). However, females had a significantly higher spine density compared to males (1.61 ± 0.039 vs. 1.39 ± 0.035 spines/µm^2^, *p* < 0,0001, *t*‐test; [Fig. 2(A,C)]). This result was mirrored in our presynaptic bouton analysis as no difference in size was noted between males and females (0.22 ± 0.02 vs. 0.26 ± 0.01 µm^2^, *p* = 0.14, *t*‐test; [Fig. 3(A,B)]), but a higher density was observed in females compared to males (42.4 ± 1.7 vs. 31.1 ± 1.8 boutons/µm^2^, *p* < 0.0001, *t*‐test; [Fig. 3(A,C)]). To test whether the increased synaptic structures observed in females were representative of functional synapses, we used double transgenic mice (*Emx1*::Cre crossed with *RC*::PSiT; Niederkofler et al., 2016) in which synaptophysin, a protein associated with synaptic vesicles at the active zone of excitatory synapses, is fused with GFP. Consistent with our previous results, measurement of GFP intensity (male 25.7 ± 1.3 a.u. vs. female 31.8 ± 1.7 a.u., *p* = 0.009, *t*‐test, [Fig. 4(A,B)]) and GFP+ puncta density revealed a significant increase in functional, synaptophysin‐containing boutons in females compared to males (3.2 ± 0.2 vs. 2.3 ± 0.15 boutons/µm^3^, *p* = 0.0005, *t*‐test, [Fig. 4(A,C)]). Taken together, these results show a higher density of synapses in the developing female hippocampus when compared to males.

### No Sex Difference in Synapse Density in the Adolescent Brain

**Figure 5:**
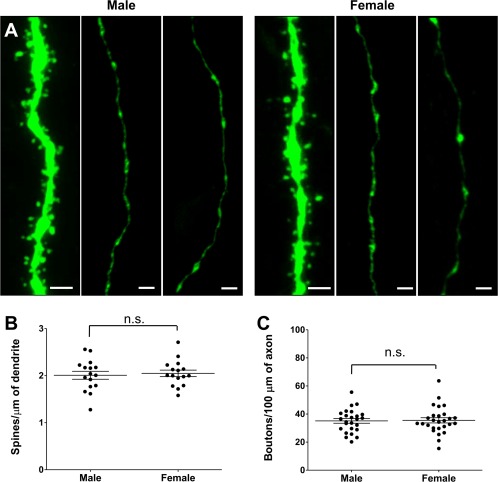
No sex difference in synaptic spine and bouton density at P40. (**A**) Representative images of GFP+ CA1 dendrites and CA3‐CA1 axons in *Thy1*::GFP males and females at P40. (**B**) Spine density measurements revealed no difference between males and females (*n* = 16 neurons from three animals for both sexes (**C**) Bouton density measurements revealed no difference between males and females (*n* = 25 and 27 axons from three animals for both sexes). The data are presented as mean ± SEM. Scale bar = 2 µm. [Color figure can be viewed at http://wileyonlinelibrary.com]

To test whether the sex differences in synapse density observed during the second postnatal week persisted in adolescence, we analyzed the density of synaptic spines and boutons in the hippocampus of *Thy1*::GFP animals at P40. We found no differences between males and females in the density of postsynaptic (2.25 ± 0.09 vs. 2.22 ± 0.11 spines/µm^2^, respectively, *p* = 0.08, *t*‐test; [Fig. 5(A,B)]) or presynaptic (38.1 ± 1.7 vs. 34.5 ± 1.3 boutons/µm^2^, respectively, *p* = 0.11, *t*‐test; [Fig. 5(A,C)]) structures in the adolescent brain, showing that the observed sex difference in synapse density at P15 is transient.

## DISCUSSION

In this study, we investigated sexual dimorphism in microglia morphology and phagocytic capacity as well as synaptic spine and bouton density and morphology in the postnatal mouse hippocampus. We found a transient peak in microglia volume and phagocytic capacity (CD68 immunocolocalization) at P15 with females demonstrating an earlier rise and fall in these features when compared to males (Fig. 1). The peak of microglia volume and phagocytic capacity was associated with a sex difference in synaptic spine and bouton density, but not size (Figs. 2, 3, 4). Notably, sex‐dependent differences in microglia and synapse density were transient and disappeared at P40 (Fig. 5).

Our data are consistent with and extend a growing body of evidence supporting sex differences in microglia morphology (Schwarz et al., 2012; Lenz et al. 2013; Hanamsagar et al. 2017; Nelson et al., 2017). The earlier rise and fall of microglia volume and phagocytic capacity in females suggest that the morphological and functional development of microglia is precocious in this sex. This hypothesis echoes a recent study that reported faster maturation of microglia morphology and gene‐expression pattern in the female hippocampus (Hanamsagar et al., 2017). Although the parallel sex‐dependent microglia and synapse differences we observe across postnatal development are strictly correlational, a growing number of studies suggest there may be a link between microglia function and circuit maturation during development. A recent time‐lapse imaging study showed that microglia can induce spine filopodia formation in the cortex during early postnatal development (Miyamoto et al., 2016), confirming the previously reported synaptogenic function of microglia during learning (Parkhurst et al., 2013). Moreover, microglia were shown to be involved in the establishment of sex‐specific synapse density in the sexually dimorphic preoptic area and associated sexual behaviors (Lenz et al, 2013).

In the present study we demonstrate transient sex differences in synapse density at P15 in the female hippocampus that have, to the best of our knowledge, not been previously reported. Although these differences did not persist at P40, it is possible that sex differences in the formation or elimination of synapses could induce sex‐specific wiring of the hippocampus. For example, in mice lacking the microglial chemokine receptor, Cx3cr1, a transient increase of synapse density in the hippocampus at P15 was recently associated with persistent deficits in the formation of multiple synapse boutons and gross brain wiring in adulthood (Zhan et al., 2014). Numerous data suggest that the hippocampus is sexually dimorphic, with sex differences reported in neurotransmitter activity, synaptic function, and hippocampus‐dependent behaviors such as spatial learning (Cahill, 2006; Bettis and Jacobs, 2009) leaving open the possibility that the transient differences we reported underlie persistent sex‐dependent effects in the adult hippocampus.

Regardless of their causal relationship, the microglia and synaptic sex differences we observe reveal a hitherto unappreciated sexual dimorphism in hippocampal development. Our work serves to underline the importance of including sex as a variable in studies of circuit maturation and confirms the presence of prominent sex differences in microglia function in the developing brain. We speculate that these differences may be important risk factors for microglia‐associated neurodevelopmental processes that affect risk for mental and neurological disorders with prominent gender biases.

Funding was provided by EMBL (C.T.G. and L.W.); U.N. was funded by People Programme (Marie Curie Actions) of the European Union's Seventh Framework Programme FP7/2007–2013/under REA grant agreement n°327409, relevant S.D. support from NIH R01 DA034022 and GVRK Khodadad Fund.
